# Supplementary figures and images for: High throughput transcriptomics analysis of ovine mammary epithelial cells stimulated with Staphylococcus aureus in vitro
Source: PLoS One. 2025 Sep 30;20(9):e0333355. doi: 10.1371/journal.pone.0333355 (PMC12483224; doi:10.1371/journal.pone.0333355)

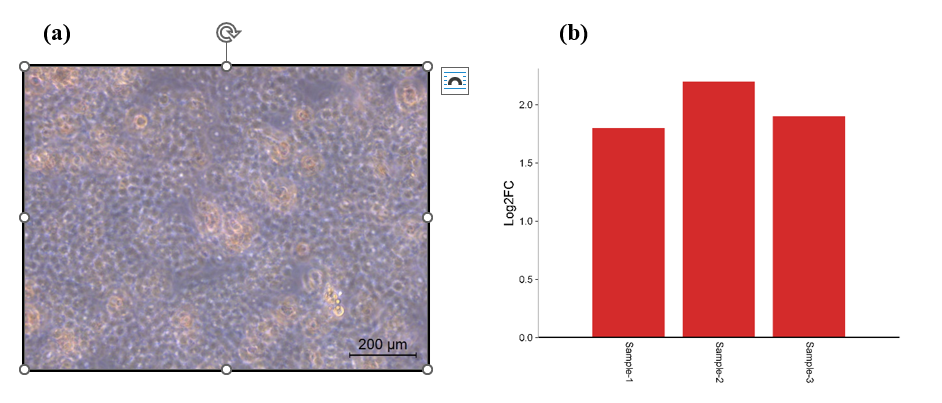

Supplement: S1 Fig — (a) Optical microscope image of cells at 200 μm scale (b) The expression of EpCAM gene, the surface cell marker of mammary epithelial cells. (TIF) [file pone.0333355.s001.tif]

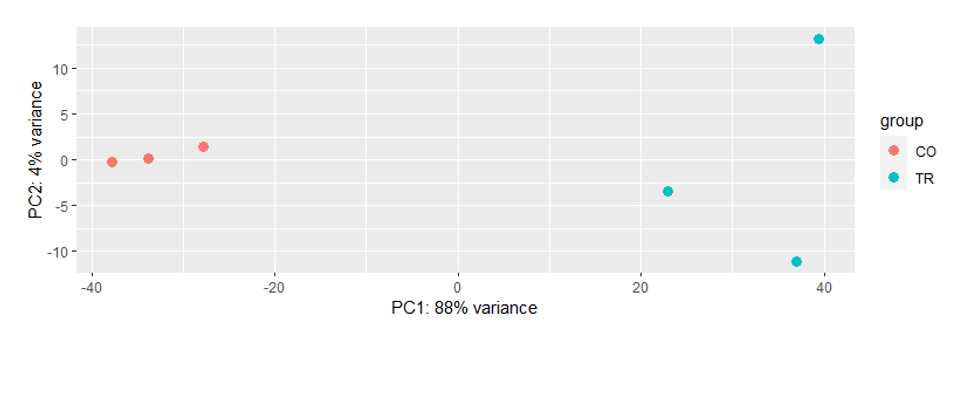

Supplement: S2 Fig — (TIF) [file pone.0333355.s002.tif]

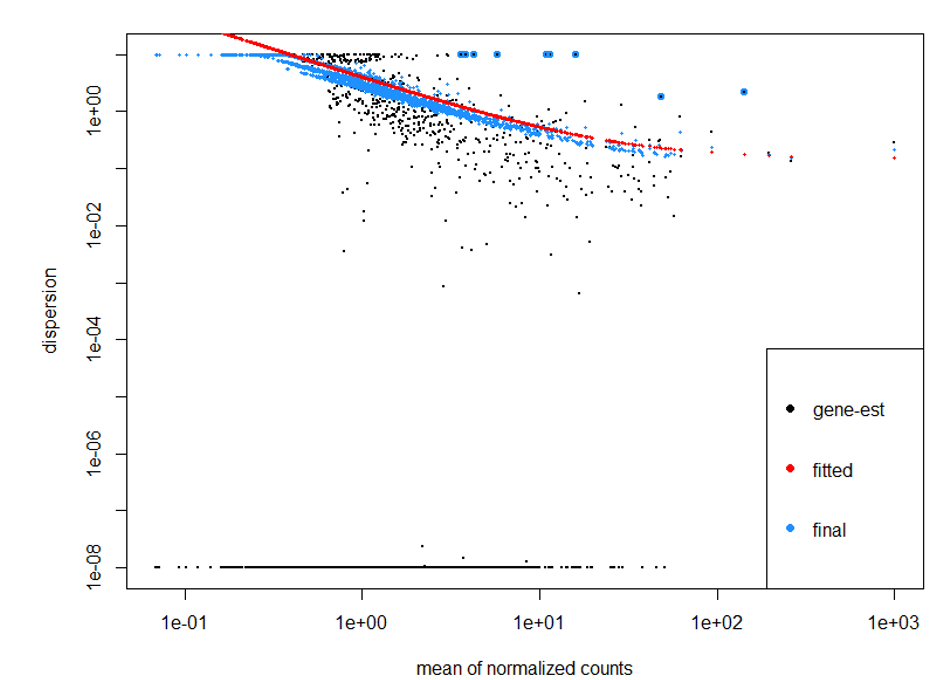

Supplement: S3 Fig — (TIF) [file pone.0333355.s003.tif]

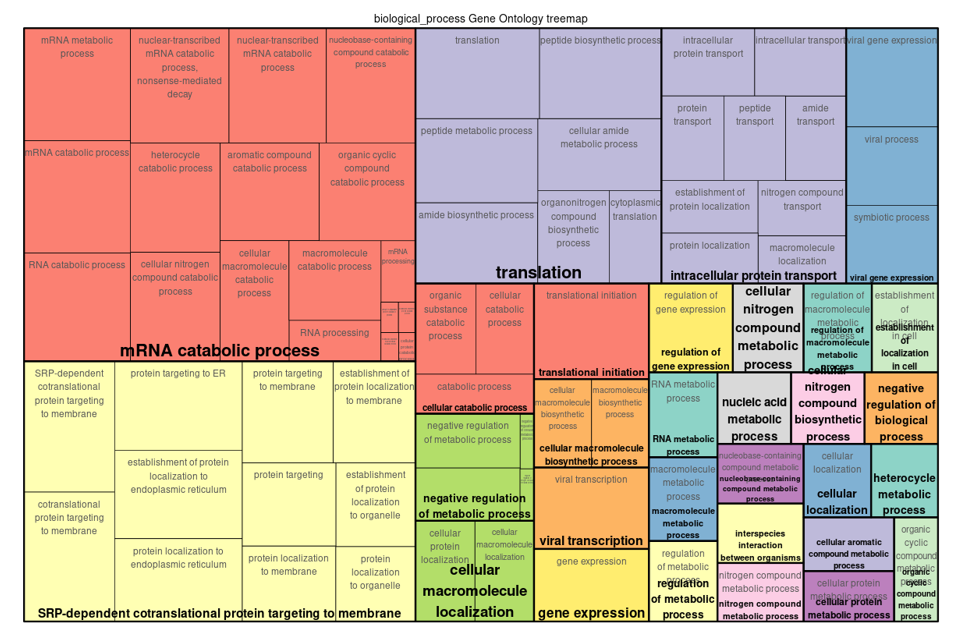

Supplement: S4 Fig — The boxes are organized into clusters corresponding to the upper hierarchy GO-term which are highlighted in bold letters. (TIF) [file pone.0333355.s004.tif]

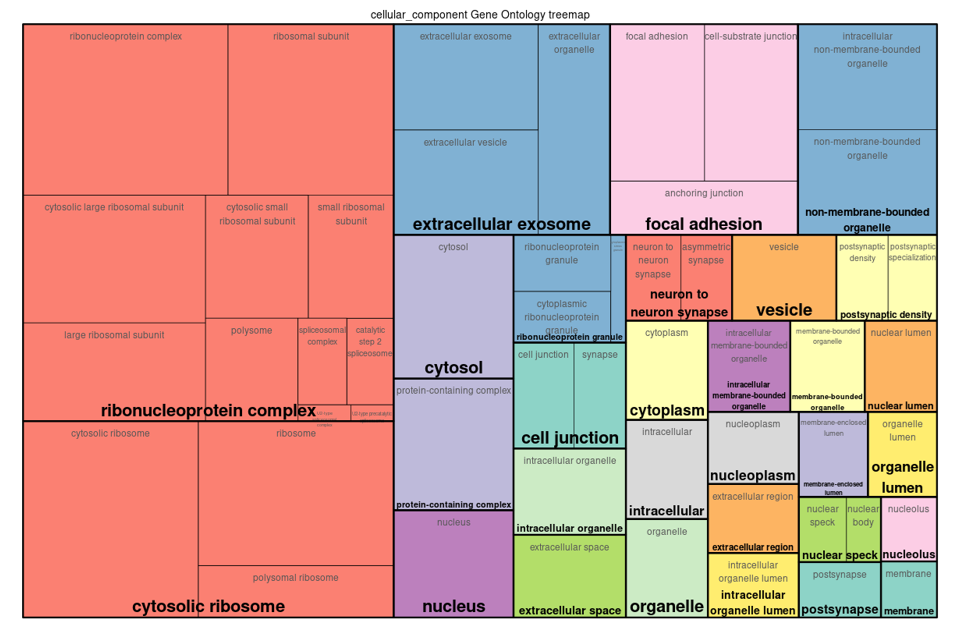

Supplement: S5 Fig — The boxes are organized into clusters corresponding to the upper hierarchy GO-term which are highlighted in bold letters. (TIF) [file pone.0333355.s005.tif]
