# Supplementary material for: High throughput transcriptomics analysis of ovine mammary epithelial cells stimulated with Staphylococcus aureus in vitro
Source: PLoS One. 2025 Sep 30;20(9):e0333355. doi: 10.1371/journal.pone.0333355 (PMC12483224; doi:10.1371/journal.pone.0333355)
Supplement: S1 Table — (DOCX) [file pone.0333355.s006.docx]

**Table S1 List of primers used for qRT-PCR**

| **Gene** | **Accession number** | **Primer sequences (5' > 3')** | **Annealing temp. (°C)** |
| --- | --- | --- | --- |
| *NEFM* | NC_056055.1 | **F-**AGGAAGGAGAAACAGAGGCT  **R-**TTTGGAGCCACTTCTTCAGC | 55 |
| *hnRNPL* | GAAI01006198.1 | **F-**TGGTGGAGTTTGAAGATGTGT  **R-**GGAGATTTTCTGGCTGGTAGA | 57 |
| *hnRNPH1* | GAAI01006968.1 | **F-**TGGGGCTCAAGGTATTCGTT  **R-**TCAGGGCCAATTTGACTTCA | 56 |
| *hnRNPF* | GAAI01000117.1 | **F-**TGCAGAATTTCCTCTCCGACT  **R-**TCAACAAAAGCCTCACCACTC | 57 |
| *DDX5* | GAAI01006136.1 | **F-**CATGGATGTGATTGCAAGGC  **R-**TCCCAGATCCAGTTTGTGCT | 58 |
| *hnRNPDL* | GAAI01004148.1 | **F-**AGTGCCTATGGTGGTGATCA  **R-**AGTCTGCATATCCCTGTCCA | 57 |
