# Supplementary material for: High throughput transcriptomics analysis of ovine mammary epithelial cells stimulated with Staphylococcus aureus in vitro
Source: PLoS One. 2025 Sep 30;20(9):e0333355. doi: 10.1371/journal.pone.0333355 (PMC12483224; doi:10.1371/journal.pone.0333355)
Supplement: S2 Table — (DOCX) [file pone.0333355.s007.docx]

**Table S2 List of differentially expressed genes as compared to control (non-stimulated) ovine epithelial cells with *S. aureus in vitro***

| **Gene ID** | **Gene description** | **Log2FC** | ***p*-value** | **Expression** |
| --- | --- | --- | --- | --- |
| ACTB | Actin Beta | 3.61 | 3.16E-08 | upregulated |
| ACTG1 | MedlinePlus Genetics | 2.87 | 0.000225369 | upregulated |
| ARF6 | ADP Ribosylation Factor 6 | 4.05 | 0.000996305 | upregulated |
| ARPC1B | Actin Related Protein 2/3 Complex Subunit 1B | 5.47 | 0.000222687 | upregulated |
| ARPC2 | Actin Related Protein 2/3 Complex Subunit 2 | 4.56 | 0.000178539 | upregulated |
| ATP5F1B | ATP Synthase F1 Subunit Beta | 4.63 | 0.003599288 | upregulated |
| BCLAF1 | BCL2 Associated Transcription Factor | 4.64 | 0.003723415 | upregulated |
| BTF3 | Basic Transcription Factor 3 | 5.83 | 3.29E-05 | upregulated |
| BTG1 | BTG Anti-Proliferation Factor 1 | 5.04 | 1.13E-05 | upregulated |
| CAPZB | Capping Actin Protein of Muscle Z-Line Subunit Beta | 4.87 | 7.19E-05 | upregulated |
| CDC42 | Cell Division Cycle 42 | 4.33 | 0.000693877 | upregulated |
| CELF1 | CUGBP Elav-Like Family Member 1 | 4.47 | 0.006026454 | upregulated |
| CELF2 | CUGBP Elav-Like Family Member 2 | 4.69 | 0.003152646 | upregulated |
| CFL1 | Gene - Cofilin 1 | 3.83 | 0.000105049 | upregulated |
| CHD3 | chromodomain helicase DNA binding protein 3 | 4.87 | 0.000186135 | upregulated |
| CNOT1 | CCR4-NOT Transcription Complex Subunit 1 | 3.55 | 0.004025434 | upregulated |
| CNOT2 | CCR4-NOT Transcription Complex Subunit 2 | 4.50 | 0.005395306 | upregulated |
| CSDE1 | Gene - Cold Shock Domain Containing E1 | 4.04 | 0.000281877 | upregulated |
| CSNK1A1 | Casein kinase 1 alpha 1 | 3.94 | 0.002443274 | upregulated |
| CTNNB1 | Catenin Beta 1 | 5.44 | 1.47E-05 | upregulated |
| CYRIB | CYFIP Related Rac1 Interactor B | 6.02 | 8.53E-08 | upregulated |
| DDX17 | DEAD-Box Helicase 17 | 4.01 | 0.001826537 | upregulated |
| DDX3X | DEAD-box helicase 3 X-linked | 5.66 | 3.69E-06 | upregulated |
| DDX5 | DEAD-box helicase 5 | 2.69 | 0.001954954 | upregulated |
| DHX15 | DEAH-Box Helicase 15 | 3.72 | 0.004748611 | upregulated |
| EEF1A1 | Eukaryotic Translation Elongation Factor 1 Alpha 1 | 5.76 | 4.18E-05 | upregulated |
| EEF1G | Eukaryotic Translation Elongation Factor 1 Gamma | 5.29 | 2.80E-06 | upregulated |
| EEF2 | eukaryotic translation elongation factor 2 | 3.14 | 4.09E-06 | upregulated |
| EIF3H | Eukaryotic Translation Initiation Factor 3 Subunit H | 3.70 | 0.005092459 | upregulated |
| EIF3M | Eukaryotic translation initiation factor 3, subunit M | 4.57 | 0.00411464 | upregulated |
| EIF4A2 | Eukaryotic Translation Initiation Factor 4A2 | 3.59 | 0.00284159 | upregulated |
| EIF4G2 | Eukaryotic translation initiation factor 4 gamma 2 | 2.53 | 0.000323656 | upregulated |
| EIF5A | Eukaryotic initiation factor 5A | 5.98 | 4.56E-07 | upregulated |
| FAU | FAU Ubiquitin Like and Ribosomal Protein S30 Fusion | 3.91 | 0.00055024 | upregulated |
| FOXP1 | Forkhead box protein P1 | 4.03 | 0.00555667 | upregulated |
| H2AZ1 | H2A. Z Variant Histone 1 | 4.90 | 0.000156807 | upregulated |
| H3F3A | H3.3 Histone A | 5.64 | 4.49E-07 | upregulated |
| HECTD1 | HECT Domain E3 Ubiquitin Protein Ligase 1 | 4.63 | 0.003374503 | upregulated |
| HNRNPA2B1 | Heterogeneous Nuclear Ribonucleoprotein A2/B1 | 5.62 | 1.60E-08 | upregulated |
| HNRNPA3 | Heterogeneous Nuclear Ribonucleoprotein A3 | 3.20 | 3.64E-05 | upregulated |
| HNRNPAB | Heterogeneous Nuclear Ribonucleoprotein A/B | 5.21 | 0.000529288 | upregulated |
| HNRNPD | Heterogeneous Nuclear Ribonucleoprotein D | 3.63 | 0.000719793 | upregulated |
| HNRNPDL | Heterogeneous Nuclear Ribonucleoprotein D Like | 4.30 | 0.001728536 | upregulated |
| HNRNPF | Heterogeneous Nuclear Ribonucleoprotein F | 6.56 | 7.55E-09 | upregulated |
| HNRNPH1 | Heterogeneous Nuclear Ribonucleoprotein H1 | 3.52 | 0.002251056 | upregulated |
| HNRNPK | Heterogeneous Nuclear Ribonucleoprotein K | 6.07 | 1.29E-05 | upregulated |
| HNRNPL | Heterogeneous Nuclear Ribonucleoprotein L | 5.94 | 6.75E-07 | upregulated |
| HNRNPM | Heterogeneous Nuclear Ribonucleoprotein M | 4.66 | 0.000514694 | upregulated |
| HNRNPU | Heterogeneous Nuclear Ribonucleoprotein U | 3.69 | 7.62E-05 | upregulated |
| HSP90AB1 | Heat Shock Protein 90 Alpha Family Class B Member 1 | 3.63 | 0.003721112 | upregulated |
| HSP90B1 | Heat Shock Protein 90 Beta Family Member 1 | 3.99 | 0.00529353 | upregulated |
| HUWE1 | ECT, UBA, and WWE Domain Containing E3 Ubiquitin Protein Ligase 1 | 3.39 | 0.002273063 | upregulated |
| KMT2E | Lysine Methyltransferase 2E (Inactive) | 4.35 | 0.001578504 | upregulated |
| LCP1 | Lymphocyte Cytosolic Protein 1 | 4.60 | 0.004514228 | upregulated |
| LDB1 | LIM Domain Binding 1 | 4.23 | 0.00060417 | upregulated |
| LOC100037668 | Novel undescribed gene | 4.52 | 0.000864585 | upregulated |
| LOC101103351 | Novel undescribed gene | 3.51 | 2.19E-08 | upregulated |
| LOC101104501 | Novel undescribed gene | 5.48 | 0.000282924 | upregulated |
| LOC101106550 | Novel undescribed gene | 4.69 | 7.45E-05 | upregulated |
| LOC101107597 | Novel undescribed gene | 4.09 | 0.003611672 | upregulated |
| LOC101108033 | Novel undescribed gene | 6.08 | 9.55E-06 | upregulated |
| LOC101108561 | Novel undescribed gene | 6.73 | 4.00E-07 | upregulated |
| LOC101108931 | Novel undescribed gene | 5.77 | 3.98E-05 | upregulated |
| LOC101109417 | Novel undescribed gene | 6.02 | 1.26E-05 | upregulated |
| LOC101109680 | Novel undescribed gene | 5.27 | 0.00033031 | upregulated |
| LOC101109899 | Novel undescribed gene | 3.32 | 0.004670068 | upregulated |
| LOC101111229 | Novel undescribed gene | 4.03 | 2.11E-09 | upregulated |
| LOC101113072 | Novel undescribed gene | 5.27 | 0.000415974 | upregulated |
| LOC101114018 | Novel undescribed gene | 3.57 | 1.97E-06 | upregulated |
| LOC101115037 | Novel undescribed gene | 5.49 | 0.000131694 | upregulated |
| LOC101115593 | Novel undescribed gene | 5.74 | 4.65E-05 | upregulated |
| LOC101116863 | Novel undescribed gene | 6.60 | 9.65E-07 | upregulated |
| LOC101117395 | Novel undescribed gene | 3.83 | 0.000400138 | upregulated |
| LOC101118481 | Novel undescribed gene | 3.51 | 0.000504027 | upregulated |
| LOC101120236 | Novel undescribed gene | 6.79 | 3.64E-07 | upregulated |
| LOC101121718 | Novel undescribed gene | 4.32 | 2.95E-08 | upregulated |
| LOC101122127 | Novel undescribed gene | 4.88 | 0.000177499 | upregulated |
| LOC101123533 | Novel undescribed gene | 6.70 | 5.08E-07 | upregulated |
| LOC105601979 | Novel undescribed gene | 7.36 | 2.53E-10 | upregulated |
| LOC105608827 | Novel undescribed gene | 6.35 | 1.02E-07 | upregulated |
| LOC105614819 | Novel undescribed gene | 3.49 | 0.005156012 | upregulated |
| LOC106990101 | Novel undescribed gene | 3.20 | 0.002921409 | upregulated |
| LOC106990434 | Novel undescribed gene | 7.06 | 2.60E-09 | upregulated |
| LOC106991051 | Novel undescribed gene | 6.31 | 4.33E-06 | upregulated |
| LOC114109518 | Novel undescribed gene | 5.61 | 8.28E-05 | upregulated |
| LOC114109527 | Novel undescribed gene | 4.77 | 0.002122948 | upregulated |
| LOC114109996 | Novel undescribed gene | 5.83 | 1.41E-07 | upregulated |
| LOC114110581 | Novel undescribed gene | 4.71 | 0.002900713 | upregulated |
| LOC114110653 | Novel undescribed gene | 5.15 | 0.000582412 | upregulated |
| LOC114112490 | Novel undescribed gene | -3.04 | 2.12E-08 | downregulated |
| LOC114112704 | Novel undescribed gene | -3.24 | 6.72E-05 | downregulated |
| LOC114112903 | Novel undescribed gene | 6.73 | 5.64E-07 | upregulated |
| LOC114113987 | Novel undescribed gene | 2.61 | 0.001962389 | upregulated |
| LOC114114600 | Novel undescribed gene | 4.40 | 3.34E-08 | upregulated |
| LOC121816057 | Novel undescribed gene | 3.95 | 0.001084968 | upregulated |
| MBNL1 | Muscleblind Like Splicing Regulator 1 | 3.94 | 1.62E-05 | upregulated |
| MORF4L1 | Mortality Factor 4 Like 1 | 4.99 | 0.000946215 | upregulated |
| NEFM | Neurofilament Medium Chain | -5.92 | 0.005088274 | downregulated |
| NPM1 | Nucleophosmin 1 | 4.28 | 0.000136472 | upregulated |
| PCBP1 | Poly (RC) Binding Protein 1 | 5.84 | 3.54E-05 | upregulated |
| PFN1 | Profilin 1 | 4.08 | 1.18E-07 | upregulated |
| PGK1 | Phosphoglycerate Kinase 1 | 5.09 | 0.000651233 | upregulated |
| PICALM | Phosphatidylinositol Binding Clathrin Assembly Protein | 4.54 | 0.004503431 | upregulated |
| PIK3CD | Phosphatidylinositol-4,5-Bisphosphate 3-Kinase Catalytic Subunit Delta | 3.96 | 0.003743527 | upregulated |
| PPIA | Peptidylprolyl Isomerase A | 5.80 | 4.19E-05 | upregulated |
| PPP1CC | Protein Phosphatase 1 Catalytic Subunit Gamma | 4.61 | 0.003950811 | upregulated |
| PPP2CA | Protein Phosphatase 2 Catalytic Subunit Alpha | 4.99 | 0.001136726 | upregulated |
| PRPF8 | Pre-MRNA Processing Factor 8 | 3.68 | 0.005162374 | upregulated |
| PSMA6 | Proteasome 20S Subunit Alpha 6 | 4.57 | 0.004261291 | upregulated |
| PTMA | Prothymosin Alpha | 4.34 | 0.001639199 | upregulated |
| PTP4A2 | Protein Tyrosine Phosphatase 4A2 | 3.17 | 0.005281092 | upregulated |
| PUM2 | Pumilio RNA Binding Family Member 2 | 3.76 | 0.004371597 | upregulated |
| RACK1 | Receptor For Activated C Kinase 1 | 4.94 | 1.49E-07 | upregulated |
| RAP1A | RAP1A, Member of RAS Oncogene Family | 5.33 | 6.60E-07 | upregulated |
| RHOA | Ras Homolog Family Member A | 4.81 | 0.000527715 | upregulated |
| RPL10L | Ribosomal Protein L10 Like | 3.74 | 0.003344745 | upregulated |
| RPL11 | Ribosomal Protein L11 | 5.00 | 1.95E-05 | upregulated |
| RPL13 | Ribosomal Protein L13 | 6.32 | 3.36E-06 | upregulated |
| RPL14 | Ribosomal Protein L14 | 5.64 | 3.73E-07 | upregulated |
| RPL15 | Ribosomal Protein L15 | 6.02 | 1.29E-05 | upregulated |
| RPL19 | Ribosomal Protein L19 | 4.13 | 2.49E-06 | upregulated |
| RPL22 | Ribosomal Protein L22 | 5.34 | 0.000318575 | upregulated |
| RPL23 | Ribosomal Protein L23 | 7.36 | 1.83E-08 | upregulated |
| RPL24 | Ribosomal Protein L24 | 4.57 | 5.26E-07 | upregulated |
| RPL26 | Ribosomal Protein L26 | 7.13 | 5.62E-08 | upregulated |
| RPL27 | Ribosomal Protein L27 | 5.27 | 0.000976887 | upregulated |
| RPL29 | Ribosomal Protein L29 | 5.62 | 0.000105791 | upregulated |
| RPL30 | Ribosomal Protein L30 | 5.43 | 1.59E-07 | upregulated |
| RPL32 | Ribosomal Protein L32 | 6.63 | 2.90E-08 | upregulated |
| RPL35 | Ribosomal Protein L35 | 5.03 | 0.001228531 | upregulated |
| RPL36AL | Ribosomal Protein L36a Like | 4.49 | 0.005417075 | upregulated |
| RPL37 | Ribosomal Protein L37 | 4.56 | 9.82E-06 | upregulated |
| RPL4 | Ribosomal Protein L4 | 5.39 | 2.82E-10 | upregulated |
| RPL6 | Ribosomal Protein L6 | 4.94 | 0.001210498 | upregulated |
| RPL7A | Ribosomal Protein L7a | 4.75 | 0.00028284 | upregulated |
| RPLP0 | Ribosomal Protein Lateral Stalk Subunit P0 | 3.65 | 1.14E-07 | upregulated |
| RPLP1 | Ribosomal Protein Lateral Stalk Subunit P1 | 3.63 | 0.00403765 | upregulated |
| RPS10 | Ribosomal Protein S10 | 5.65 | 6.61E-05 | upregulated |
| RPS13 | Ribosomal Protein S13 | 6.05 | 1.08E-05 | upregulated |
| RPS14 | Ribosomal Protein S14 | 4.43 | 1.04E-10 | upregulated |
| RPS15 | Ribosomal Protein S15 | 6.78 | 3.22E-07 | upregulated |
| RPS18 | Ribosomal Protein S18 | 4.30 | 3.27E-07 | upregulated |
| RPS19 | Ribosomal Protein S19 | 5.23 | 1.82E-10 | upregulated |
| RPS2 | Ribosomal Protein S2 | 7.73 | 1.83E-09 | upregulated |
| RPS23 | Ribosomal Protein S23 | 7.98 | 4.99E-10 | upregulated |
| RPS24 | Ribosomal Protein S24 | 3.28 | 2.69E-05 | upregulated |
| RPS25 | Ribosomal Protein S25 | 5.42 | 0.000207722 | upregulated |
| RPS26 | Ribosomal Protein S26 | 5.07 | 0.000704871 | upregulated |
| RPS27 | Ribosomal Protein S27 | 5.57 | 7.57E-07 | upregulated |
| RPS27A | Ribosomal Protein S27a | 3.65 | 0.000237091 | upregulated |
| RPS3 | Ribosomal Protein S3 | 6.61 | 8.19E-07 | upregulated |
| RPS7 | Ribosomal Protein S7 | 5.06 | 8.29E-05 | upregulated |
| RPS8 | Ribosomal Protein S8 | 4.55 | 9.92E-09 | upregulated |
| RPS9 | Ribosomal Protein S9 | 3.15 | 0.004060838 | upregulated |
| RPSA | Ribosomal Protein SA | 3.84 | 3.78E-07 | upregulated |
| SATB1 | SATB Homeobox 1 | 4.88 | 0.001790157 | upregulated |
| SERBP1 | SERPINE1 MRNA Binding Protein 1 | 3.30 | 0.000169529 | upregulated |
| SERF2 | Small EDRK-Rich Factor 2 | 5.77 | 4.06E-05 | upregulated |
| SF3B1 | Splicing Factor 3b Subunit 1 | 4.78 | 5.28E-05 | upregulated |
| SNX5 | Sorting Nexin 5 | 4.11 | 0.003274551 | upregulated |
| SPTBN1 | Spectrin Beta, Non-Erythrocytic 1 | 5.04 | 0.00113956 | upregulated |
| SRSF1 | Serine And Arginine Rich Splicing Factor 1 | 3.87 | 0.000974344 | upregulated |
| SRSF2 | Serine And Arginine Rich Splicing Factor 2 | 3.71 | 0.002702057 | upregulated |
| TPM3 | Ropomyosin 3 | 4.44 | 0.001860991 | upregulated |
| TUBB | Tubulin Beta Class I | 5.97 | 2.66E-05 | upregulated |
| UBA52 | Ubiquitin A-52 Residue Ribosomal Protein Fusion Product 1 | 5.59 | 0.000148367 | upregulated |
| UBB | Ubiquitin B | 3.15 | 9.71E-06 | upregulated |
| UBC | Ubiquitin C | 3.24 | 0.000112634 | upregulated |
| UBE2D3 | Ubiquitin Conjugating Enzyme E2 D3 | 3.00 | 0.001289603 | upregulated |
| UBE2Q1 | Ubiquitin Conjugating Enzyme E2 Q1 | 4.48 | 0.006337563 | upregulated |
| UBR4 | Ubiquitin Protein Ligase E3 Component N-Recognin 4 | 4.11 | 0.003937385 | upregulated |
| XRN2 | 5'-3' Exoribonuclease 2 | 4.27 | 0.001764769 | upregulated |
| YBX1 | Y-Box Binding Protein 1 | 3.25 | 2.29E-05 | upregulated |
| YWHAE | Tyrosine 3-Monooxygenase/Tryptophan 5-Monooxygenase Activation Protein Epsilon | 3.99 | 0.000804131 | upregulated |
| YWHAZ | Protein Codin | 4.56 | 0.000115124 | upregulated |
