# Supplementary material for: High throughput transcriptomics analysis of ovine mammary epithelial cells stimulated with Staphylococcus aureus in vitro
Source: PLoS One. 2025 Sep 30;20(9):e0333355. doi: 10.1371/journal.pone.0333355 (PMC12483224; doi:10.1371/journal.pone.0333355)
Supplement: Attachment — Submitted filename: Response to Reviewers.docx [file pone.0333355.s010.docx]

**Point-by-point response**

**High throughput transcriptomics analysis of ovine mammary epithelial cells stimulated with *Staphylococcus aureus in vitro***

***Academic editor***

**Comment**: The reviewers' feedback has been generally positive, and your manuscript is moving toward acceptance pending revisions. Although the reviewers have accepted the core content, I have come across some relevant and interesting papers that could further enhance the quality and depth of your work. I recommend reviewing these papers and incorporating relevant information or citations where appropriate to strengthen the manuscript.

**Response**:

Thank you for your constructive suggestions and for highlighting additional literature relevant to our study. We have carefully reviewed the papers you provided. The study by Nadi et al. (2023) examines the prevalence of *Staphylococcus aureus*, *Pseudomonas aeruginosa* and *Escherichia coli* in dairy products and reports a high frequency of methicillin resistance among *Staphylococcus aureus* isolates; this citation has been incorporated into the introduction section of manuscript to emphasize the public health significance of antimicrobial‑resistant *Staphylococcus aureus* strains. The work by Torun et al. (2025) compares virulence and resistance genes among coagulase‑negative staphylococci and *Staphylococcus aureus* from raw milk, we now reference this study in the introduction to underscore the diversity of staphylococcal species and the prevalence of the mecA gene. Zafar et al. (2025) demonstrate that *Staphylococcus aureus* is the most frequent cause of mastitis and explore the antibacterial potential of copper nanoparticles. We cite this study to highlight the alternative therapeutic strategies against mastitis. These citations strengthen the context of our research and underscore the relevance of our transcriptomic analysis. The recommended reference; Rashid et al. (2024) was not cited, as it was not relevant to the present study.

***Reviewer #4***

**Comment**: 1. The article is well-structured, clearly written, and presents a solid dataset supporting its conclusions. The section discussing differential gene expression (DEG) analysis and the immune response in ovine mammary epithelial cells (oMECs) following Staphylococcus aureus stimulation is generally well explained and informative. However, a minor revision is recommended to improve the clarity and scientific rigor of the Discussion section.

**Response**: Thank you for acknowledging our work and for your positive feedback regarding the structure, clarity and data set presented in the manuscript. We appreciate your constructive suggestion to enhance the clarity and scientific rigor of the Discussion section. In response, we have carefully revised this section to better contextualize our findings.

**Comment No 1:** 1. Line 271: The phrase "|log2 (fold change)| < 1" seems inconsistent with standard DEG filtering, as typically DEGs are considered significant if the absolute log2 fold change is greater than or equal to 1. If this is a typo, it should be corrected to "|log2 (fold change)| ≥ 1". Otherwise, the rationale for using a <1 threshold should be justified.

**Response No 1:** We thank the reviewer for pointing out this inconsistency. The phrase "|log₂ (fold change)| < 1" was indeed a typographical error. As correctly noted, the standard threshold for identifying differentially expressed genes is "|log₂ (fold change)| ≥ 1". We have reviewed the manuscript thoroughly and corrected this error to ensure consistency throughout the text

**Comment No 2:** 2. Line 272: Strengthen transitions between comparative studies. The comparisons with other studies (e.g., Chen et al., [1]) are useful but could benefit from more concise phrasing and a clearer connection to the current study's unique findings. Please see “Riaz, M. et al. (2025). Transcriptomic insights into the bovine immune response following Staphylococcus aureus infection. International Journal of Veterinary Science, 14(3), 200–208. Available at: https://www.ijvets.com/article/264/” as a suggested citation and a supporting reference to strengthen your argument.

**Response 2:** Thank you for noting that the comparative section of the discussion could be more concise. In the revised manuscript we extended the section comparing our findings with previous studies. Although the recommended study was not accessible because of incorrect information International Journal of Veterinary Science, volume 14(3) contained page 434-623 (<https://www.ijvets.com/volume-14-no-3-2025/> ). Even volume 14(2) and 14(1) does not contain any such article (<https://www.ijvets.com/volume-14-no-2-2025/>, <https://www.ijvets.com/volume-14-no-1-2025/> ).

**Comment No 3:** 3. While the discussion on high duplicate reads is relevant, it could be summarized succinctly or referenced with greater clarity regarding how it affects DEG reliability in this study.

**Response No 3:** We appreciate your suggestion to summarize the discussion of duplicate reads. We have condensed this part and clarified that the high proportion of duplicate reads in our dataset likely reflects the saturation of mapping space by highly expressed transcripts rather than technical artefacts. We also refer to existing literature to explain that removal of duplicates can bias expression estimates when duplicates originate from abundant transcripts.

**Comment No 4:** 4. The interpretation of upregulated genes such as RPS19, hnRNPs, and ACTB is commendable. Still, it could be further refined by linking their immune roles more directly to the context of S. aureus infection in oMECs. The manuscript would benefit from a deeper exploration of the immune pathways' molecular intricacies.

**Response No 4:** Thank you for encouraging a deeper exploration of the immune roles of the up‑regulated genes. We expanded the discussion to link *RPS19*, *RPS14*, *hnRNPs* and *ACTB* to specific innate immune pathways. For example, we note that RPS19 interacts with macrophage migration inhibitory factor, *ERK* and *NF‑κB*, bridging translation and inflammatory signalling; *RPS14* contributes to TLR4 recognition of *Staphylococcus aureus*. The *hnRNP* family members regulate the stability of transcripts encoding cytokines such as *TNF‑α* and *IL‑6*. Similarly, *ACTB* participates in cytoskeletal rearrangements required for pathogen internalisation and leukocyte migration. We also discuss how these genes fit into broader pathways such as TLR, NOD‑like receptor, NF‑κB and MAPK signalling, thereby providing a more comprehensive interpretation of our transcriptomic data.
